# Management of the Sick Room
*Practical Hints on the Management of the Sick Room. By R. Hall Bakewell, M.D. London: Snow. 1857.


**Published:** 1857-07

**Authors:** 


### MANAGEMENT OF THE SICK ROOM.*

This little work, by Dr. Bakewell, contains useful hints. They may be epitomised as follows:—1. If you think yourself so ill as to require advice, get it at once from a qualified man. If you require no doctor, you are better without physic. 2. Ventilate the sick room thoroughly. 3. If there is in the sick room an offensive odour, do not make it worse by disguising it, by burning pastiles or other trash—but let in the air, and let out the cause of the smell. 4. Water-beds are the most useful for the prevention of bed-sores. 5. Keep the sick man quiet—a fussy nurse is worse than a lazy one. 6. Chloride of lime is the best disinfectant.

---

* Practical Hints on the Management of the Sick Room. By R. Hall Bakewell, M.D. London: Snow. 1857.

